# Assessment of Fecal Indicator Bacteria and Potential Pathogen Co-Occurrence at a Shellfish Growing Area

**DOI:** 10.3389/fmicb.2018.00384

**Published:** 2018-03-14

**Authors:** Andrew K. Leight, Byron C. Crump, Raleigh R. Hood

**Affiliations:** ^1^Cooperative Oxford Laboratory, National Ocean Service/National Centers for Coastal Ocean Science, National Oceanic and Atmospheric Administration (NOAA), Oxford, MD, United States; ^2^Horn Point Laboratory, University of Maryland Center for Environmental Science, Cambridge, MD, United States; ^3^College of Earth, Ocean, and Atmospheric Sciences, Oregon State University, Corvallis, OR, United States

**Keywords:** microbiome, estuary, human health, shellfish closures, pathogens

## Abstract

Routine monitoring of shellfish growing waters for bacteria indicative of human sewage pollution reveals little about the bacterial communities that co-occur with these indicators. This study investigated the bacterial community, potential pathogens, and fecal indicator bacteria in 40 water samples from a shellfish growing area in the Chesapeake Bay, USA. Bacterial community composition was quantified with deep sequencing of 16S rRNA gene amplicons, and absolute gene abundances were estimated with an internal standard (*Thermus thermophilus* genomes). Fecal coliforms were quantified by culture, and *Vibrio vulnificus* and *V. parahaemolyticus* with quantitative PCR. Fecal coliforms and *V. vulnificus* were detected in most samples, and a diverse assemblage of potential human pathogens were detected in all samples. These taxa followed two general patterns of abundance. Fecal coliforms and 16S rRNA genes for Enterobacteriaceae, *Aeromonas, Arcobacter, Staphylococcus*, and *Bacteroides* increased in abundance after a 1.3-inch rain event in May, and, for some taxa, after smaller rain events later in the season, suggesting that these are allochthonous organisms washed in from land. Clostridiaceae and *Mycobacterium* 16S rRNA gene abundances increased with day of the year and were not positively related to rainfall, suggesting that these are autochthonous organisms. Other groups followed both patterns, such as *Legionella*. Fecal coliform abundance did not correlate with most other taxa, but were extremely high following the large rainstorm in May when they co-occurred with a broad range of potential pathogen groups. *V. vulnificus* were absent during the large rainstorm, and did not correlate with 16S rRNA abundances of *Vibrio* spp. or most other taxa. These results highlight the complex nature of bacterial communities and the limited utility of using specific bacterial groups as indicators of pathogen presence.

## Introduction

Monitoring for indicator microorganisms, such as fecal coliforms, *Escherichia coli*, and *Enterococcus* spp., at shellfish beds and swimming beaches has been conducted since the early 1900's to assess the likelihood of encountering human pathogens from fecal pollution (EPA, 1986; Ashbolt et al., 2001). However, the presence and abundance of these indicator bacteria are not always correlated with the presence of human pathogens (Noble and Fuhrman, 2001) and their strength in risk assessments depends on cell abundance (EPA, 1986; FDA, 2013) and the types of pathogens present (Wade et al., 2003). More reliable indicators of fecal pollution and human pathogens are being sought and several other bacterial groups have been proposed, including several members of the order Bacteriodales (Bernhard and Field, 2000; Walters et al., 2007) and the family Lachnospiraceae (Newton et al., 2011).

Several studies have explored the co-occurrence of pathogens and indicator bacteria in bivalves or their surrounding waters. For example, Walters et al. (2007) found that *Salmonella, Campylobacter*, and *E. coli* O157:H7 did not commonly co-occur in water samples, suggesting different sources or life-histories for these pathogens. While Hood et al. (1983) found that high levels of fecal coliforms and *E. coli* were about equally predictive of the presence of the specific pathogen group *Salmonella* in oyster tissue, a single indicator for the diverse types of pathogens found at shellfish beds or swimming beaches may not be feasible (Wade et al., 2003).

In the Chesapeake Bay, monitoring for indicator bacteria over natural shellfish beds occurs year-round. While the harvest of oysters from these natural beds is currently prohibited from late spring to early fall, harvest from aquaculture is permissible year-round, with additional post-harvest handling requirements during summer months (MDDNR, 2015). In Maryland, fecal indicators in waters over natural shellfish beds are monitored with measurements of fecal coliform densities twice a month. Local health agencies conduct similar routine monitoring during the summer in recreational areas of the Chesapeake Bay used for boating and swimming.

Despite these monitoring efforts, little is known about the bacterial communities that co-occur with fecal indicator bacteria. Bacterial communities in natural waters tend to be very diverse (Kan et al., 2006) and have complex and redundant functional groups (Comte and del Giorgio, 2010). Until recently, studies of bacterial communities in shellfish growing areas have relied on culture-based methods that exclude a large number of bacteria and require focused analyses of particular bacterial groups. A study in the Santa Anna River, California using 16S rRNA gene amplicon pyrosequencing detected higher percentages of genera that contain human pathogens in areas of urban runoff and agriculture (Ibekwe et al., 2013). Likewise, a recent study of bacterial community variability at a shellfish growing area in Spain, using denaturing gradient gel electrophoresis (DGGE), noted seasonal shifts in the bacterial community and the presence of genera containing human pathogens (Pereira et al., 2015). However, this study included samples from only six time points between February and December. The occurrence and residence time of bacterioplankton in rivers and estuaries can change over time periods of hours and days (Heidelberg et al., 2002; Crump et al., 2004) and the survival and abundance of human pathogens in natural waters may relate to temporal changes in water temperatures and salinities (Rhodes and Kator, 1988; Jacobs et al., 2014). Several more recent studies have used next-generation sequencing to investigate the presence of human pathogens in drinking water and to compare the occurrence of these pathogens to indicator bacteria (Inkinen et al., 2016; Shrestha et al., 2017). Therefore, additional studies using next-generation sequencing and focused on more highly resolved temporal changes in estuarine bacterial communities and potential pathogenic members at shellfish growing areas, would help inform human health risks relative to traditional indicators and potentially improve decisions about restricting shellfish harvest.

The presence of human pathogens that are not of fecal origin represent a significant concern in many estuarine areas. Pathogenic groups of *Vibrio* bacteria, such as *V. vulnificus* and *V. parahaemolyticus*, for example, are naturally-occurring and cause numerous cases of gastroenteritis and wound infections (Johnson et al., 2012). Environmental factors influencing their abundance, such as water temperature and salinity, are known (Johnson et al., 2012; Jacobs et al., 2014), but the co-occurrence of these pathogens with other bacterial members of the plankton is poorly understood.

The objectives of this study were to assess the abundances of fecal indicator bacteria and specific *Vibrio* bacteria in a shellfish growing area of the Chesapeake Bay over time, and to identify members of the bacterial community, including potential pathogen groups, that co-occur with these organisms. An additional objective was to relate the occurrence of fecal coliform and *Vibrio* species with the greater bacterial community and with potential drivers of the temporal changes detected in these taxa.

In order to address these objectives, this study used high-throughput DNA sequencing of 16S rRNA gene PCR amplicons to identify and quantify bacterial community members. High-throughput sequencing techniques typically only provide estimates of relative abundance, and not absolute abundance, but some recent studies have overcome this limitation using internal controls (Moran et al., 2013; Satinsky et al., 2013; Smets et al., 2016). Moran et al. (2013) and Satinsky et al. (2013) added a known concentration of *Thermus thermophilus* HB-8 genomes to filter-concentrated plankton samples prior to DNA extraction in order to estimate the absolute abundance of genes identified in metagenome sequence libraries. Smets et al. (2016) used a similar approach to estimate the absolute abundance of 16S rRNA genes identified in PCR amplicon libraries from soils, and found that these abundances correlated with the mass of soil extracted and the abundance of bacterial cells. We used internal controls to estimate absolute abundances of 16S rRNA genes in plankton samples in order to compare the abundance of bacterial community members identified with high-throughput sequencing, with culture-derived abundances of indicator bacteria and quantitative PCR-based abundances of specific *Vibrio* taxa.

## Materials and methods

### Water sampling

The sampling location was a long-term Maryland Department of the Environment monitoring station near the mouth of Town Creek (Figure S1), surrounded by the Town of Oxford (Maryland, USA) to the west and agricultural fields and low density residential areas to the east. The Town of Oxford manages a secondary wastewater treatment facility that releases approximately 125,000 gal/day of effluent (Scott Delude, Public Works Director, personal communication). Because of proximity to the treatment plant, shellfish harvest and aquaculture are currently forbidden in Town Creek, though routine culture-based testing of treatment plant effluent shows fecal bacteria levels below the detection limit (<1.8 MPN (Most Probable Number) per 100 mL of sample, (standard method 9221E; APHA, 1998)). Total depth at the sampling location is approximately 3.0 m, tidal amplitude at this location averages about 0.6 m (NOAA, 2015), and salinity averages 10 ppt (current study).

Water samples were collected on 14 days between April 14 and September 3, 2014, from both surface and bottom waters, and replicate samples were collected on 9 days. Water was collected in sterile polypropylene bottles (500 mL) by hand from surface waters and by Van Dorn sampler (Wildco, Inc.) from bottom waters (0.25 m above bottom), placed on ice, and processed within 30 min of collection. Water physicochemical measurements were collected with a YSI 6600 datasonde (YSI, Inc., Yellow Springs, OH). Precipitation was measured with a tipping bucket rain gauge (Onset, Inc.) stationed approximately 1.6 km from the water collection site (Figure S1).

Recovery of DNA by water filtration was performed using established methods (Fortunato and Crump, 2011). Between 250 and 300 mL of water was filtered through a 0.2 μm Sterivex-GP filter (EMD Millipore, Darmstadt, Germany), the volume filtered was recorded, and 1 mL of filter-sterilized DNA extraction buffer (DEB; 0.1M Tris-HCL (pH 8), 0.1M Na-EDTA (pH 8), 1.5M NaCl, 5% Cetyltrimethyl ammonium bromide) was added to each filter cartridge. The filters were then stored at −80°C, and later extracted according to published methods (Crump et al., 2003), including several freeze/thaw cycles and final DNA isolation by isopropanol precipitation. Prior to extraction, approximately 60 million copies of the *T. thermophilus* genome (i.e., 250 ng *T. thermophilus* DNA) were added to each sample, in order to approximate a final ratio of 1.00:0.04 natural bacterial cells to *T. thermophilus* genomes, based on expected cell abundance in Chesapeake Bay waters (Heidelberg et al., 2002; Kan et al., 2006).

### DNA sequencing

DNA library preparation and sequencing followed Fadrosh et al. (2014). A segment of the 16S rRNA gene including the V3 and V4 hypervariable regions was amplified using PCR. Amplification was confirmed by running several samples on an electrophoresis gel and checking for bands with an approximate length of 469 base pairs. Universal 16S rRNA gene primers (319F and 806R) were complemented with a heterogeneity spacer sequence of variable length and an Illumina (Illumina, Inc., San Diego, CA) index sequence. Sequencing was performed on the MiSeq platform (Illumina, Inc., San Diego, California, USA), which produced an average of 131,000 reads per sample. Processing of reads was conducted using Mothur (Schloss et al., 2009; Mothur, 2015) and following Kozich et al. (2013). All reads were archived in the Sequence Read Archive (accession #SRP073436).

A phylotypic approach (Mothur command “phylotype”) was used for clustering sequences into operational taxonomic units (OTUs), with a cutpoint of 97% similarity for classification (Mothur, 2015). Chimeric sequences were identified and removed using the function chimera.uchime. OTU representative sequences were classified by comparison to the Silva database, version 1.19 (Silva, 2015), and OTUs not classified as Eubacteria (i.e., Eukaryota, mitochondria, chloroplasts, or unclassified) were deleted. The per liter abundance of 16S rRNA gene copies per OTU in the original sample population was estimated from the number of reads of the internal control, *T. thermophilus*. A correction factor was applied to the number of reads in each sample based on the ratio of 16S rRNA genomes added to original samples compared to the number of reads for *T. thermophilus* after sequence processing (Equation 1). Although this approach does not account for gene copy number per cell or differential amplification success of different bacteria, it provided an estimate of the density of 16S rRNA genes present in the samples for comparison across samples. A similar approach was taken by Smets et al. (2016) to estimate 16S rRNA gene abundances in soil samples.

$$\begin{array}{l} \left((Tt\;genomes\text{ added})\;/\;(2\;\times \;Tt\text{ seqs}))\;\times \;(\text{non}-\;Tt\text{ seqs}\right)\\ \times \;(1\;/\text{ volume filtered})\;=\;16S\;rRNA\text{ genes }/\text{ mL} \end{array}$$

Where *Tt* = *T. thermophilus* and seqs = sequences.

### Fecal coliform and *Vibrio* abundance estimations

Fecal coliform densities were estimated using the standard three-tube culture method (APHA, 1998). Test tubes containing inverted gas-tubes and various concentrations of A1 broth were inoculated with aliquots of sample water, incubated in a 35°C water bath for 3 h (±30 min), and then in a 44.5°C water bath for 21 h (±2 h). Tubes showing turbidity and gas production were counted as positive for growth of fecal coliforms. Samples were tested for fecal coliforms on all dates except August 22 when an equipment failure compromised the cultures. An estimate of the number of fecal coliform bacteria in 100 mL of water was made by dividing the Most Probable Number (MPN) by 100.

Estimates of *V. vulnificus* (*Vv*) and *V. parahaemolyticus* (*Vp*) abundance, independent of the 16S rRNA community analysis, were conducted using quantitative PCR (qPCR) (Jacobs et al., 2014), with primers and probes adapted from Panicker and Bej (2005) and Nordstrom et al. (2007), respectively (Table S1). A unique internal control was incorporated simultaneously into the qPCR assay to test for the presence and influence of inhibitors (Nordstrom et al., 2007). The *V. vulnificus* and *V. parahaemolyticus* standard curves were developed separately using optical density and spread plate cultures to estimate the *Vibrio* cells per milliliter of water, allowing the reporting of qPCR results as *Vibrio* cells per milliliter of sample. DNA for the standard curve was filtered from the spiked water samples that were diluted for spread plating, and extracted using the same protocol as the environmental samples (described in Section Water sampling). The standard curve was included on each microplate. All qPCR assays had efficiencies between 97 and 100%, standard curve *R*^2^ values greater than 0.990, and limits of detection of 2 cells per mL (200 copies per assay, assuming one copy per cell). Each assay included a negative control that was always below the limit of detection.

### Selection of potential pathogen groups

Bacterial genera that largely contain human pathogens or include species endemic to the human gut were identified. These taxonomic groups included *Aeromonas, Arcobacter, Campylobacter, Legionella, Helicobacter, Toxoplasma*, and members of the Enterobacteriaceae (e.g., *Yersinia, Klebsiella, Escherichia, Shigella*). In addition, genera that contain common foodborne (Scallan et al., 2011) and waterborne (Pond, 2005) human pathogens, but also include non-pathogenic members, were identified. These genera included *Clostridium, Leptospira, Listeria, Mycobacterium, Staphylococcus*, and *Vibrio*. The genera *Coxiella, Rickettsia*, and *Franciscella*, were also included in our screening of 16S rRNA gene sequences because they are listed by the United States Health and Human Services Administration as important bacterial pathogens (HHS, 2017).

### Data analyses

Prior to diversity calculations, the number of sequences per sample was rarefied to 17,500. Alpha diversity measurements (Shannon Diversity and the Chao1 estimate of taxon richness) were calculated for each sample using R (The R Foundation for Statistical Computing, version 3.1.2). Beta diversity was compared between samples using Bray-Curtis Similarity matrices and plotted with non-metric multi-dimensional scaling (nMDS) using R and by cluster analysis using PRIMER for Windows (PRIMER-E Ltd, version 6). Comparison of community composition to environmental variables was conducted via distance-based linear modeling (DistLM) using PRIMER. All other comparisons involved estimated 16S rRNA gene densities. For rare taxa, with 16S rRNA genes detected in only one or two samples, presence vs. absence was compared between 16S rRNA phylotypes, fecal coliforms (culture method), *V. vulnificus* and *V. parahaemolyticus* (qPCR) estimates. For more commonly occurring potential pathogen groups (detected in three or more samples), estimated abundances of bacterial genera (16S rRNA sequences), fecal coliforms (culture method), *V. vulnificus* and *V. parahaemolyticus* (qPCR), and environmental factors (e.g., water clarity, rainfall) were compared using Spearman correlation analysis in SAS (SAS Institute, Inc.; version 9.4). Significant correlations were then compared visually using a network analysis (R, package iGraph). All statistical tests were considered significant at *p* < 0.05.

## Results

### Environmental conditions

Conditions changed seasonally and were similar between surface and bottom waters, with a small decrease in dissolved oxygen in bottom waters during May and August (Table S2). Mean water temperatures increased from 15°C in April to 26°C in June, and remained high through September. Salinity ranged from 9.1 to 11.7 ppt, with the lowest values in June and highest values in May and September. Water clarity (Secchi depth) decreased in June and remained low thereafter. Fifteen precipitation events occurred during the study, ranging from 0.05 to 1.30 inches of rain (Figure S2). Four events exceeding 0.50 inches of rain in the previous 24 h were sampled (May 1, May 28, June 13, and June 24), including one event (May 1) that exceeded 1 inch of rain.

### Bacterial community composition

**Figure 1:**
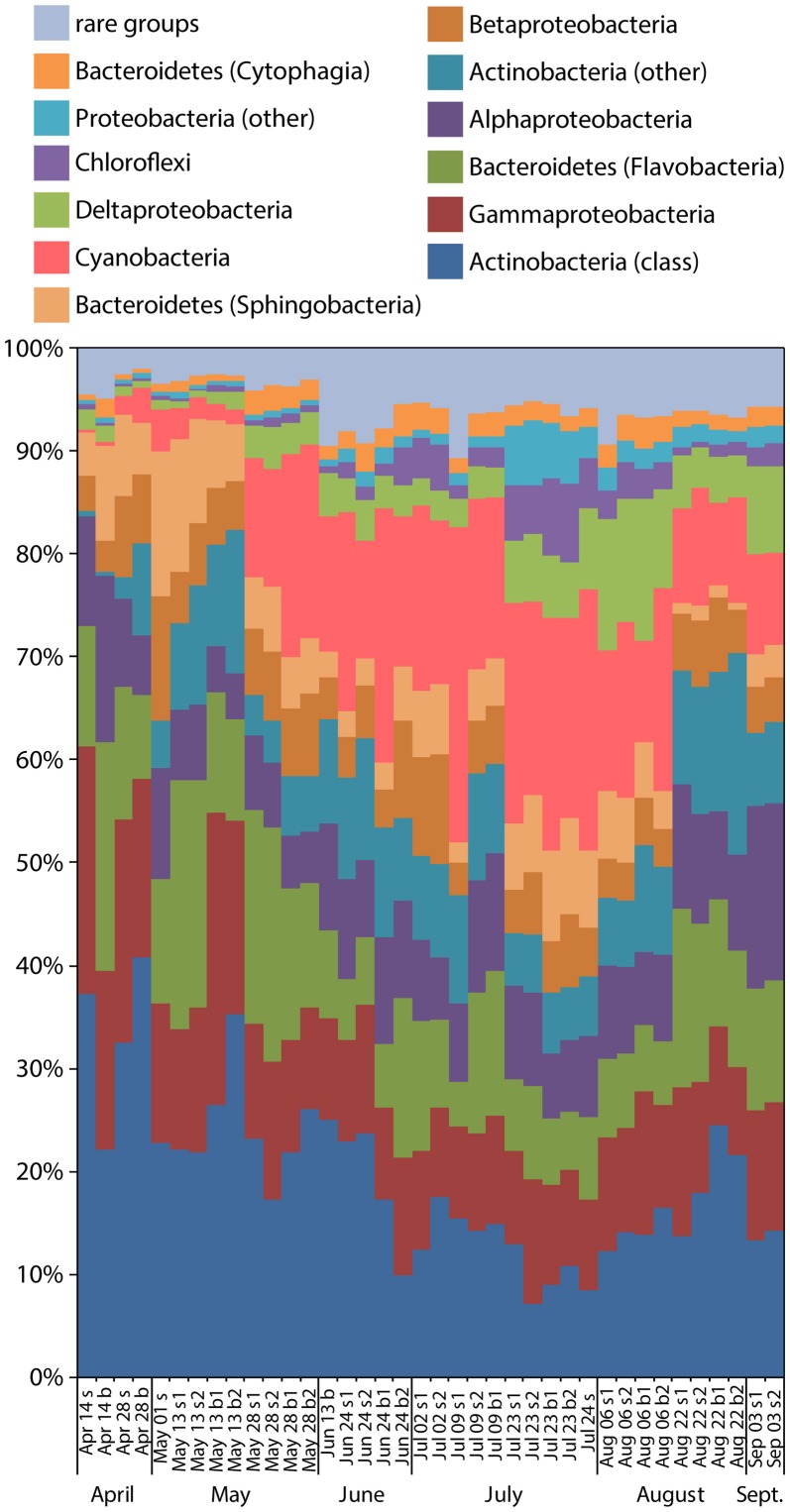
Relative abundances of 16S rRNA genes for the 12 most common taxonomic groups by sample. The phyla Proteobacteria were separated into Alpha-, Beta-, Gamma-, Delta-, and “other” classes; Bacteroidetes into Cytophagia, Sphingobacteriia, Flavobacteria, and “other” classes; and Actinobacteria into the class Actinobacteria and “other” classes.

**Figure 2:**
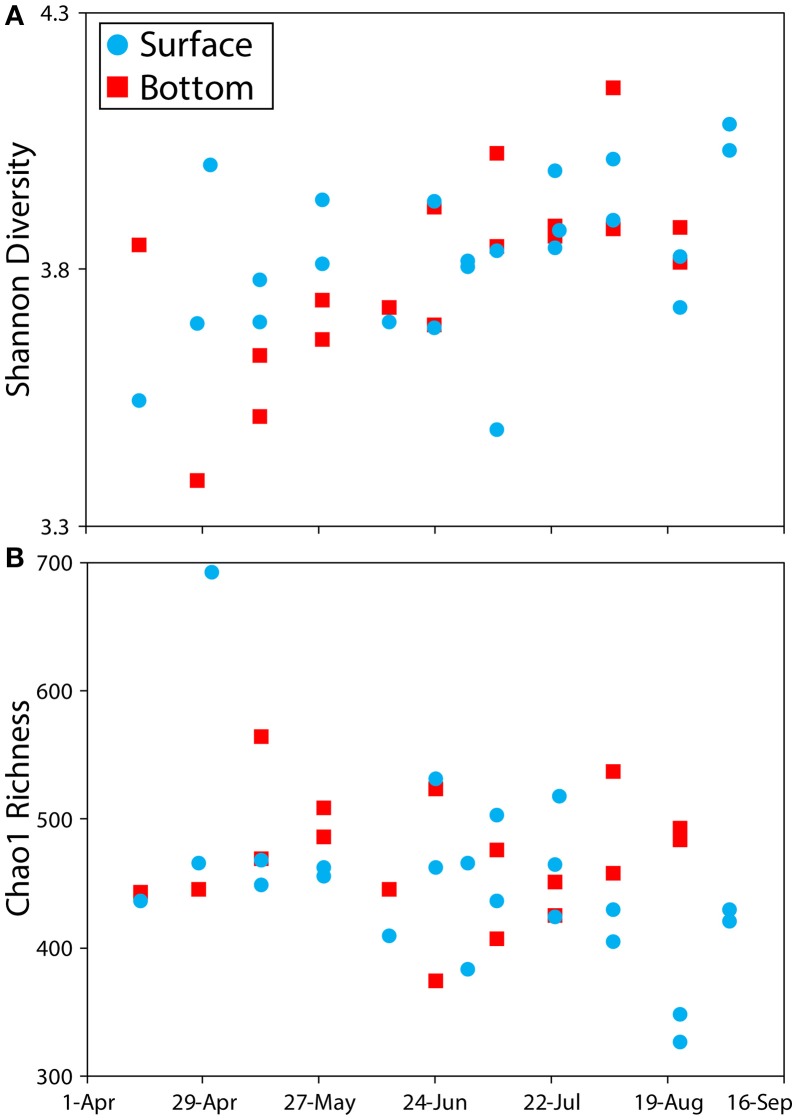
Measurements of alpha diversity by sample date and sample depth (surface and bottom). Panel **(A)** shows Shannon diversity values and panel **(B)** shows Chao1 richness.

Using the phylotypic approach to sequence classification (“phylotype” command in Mothur software package) (Schloss et al., 2009), the samples contained an average of 541 operational taxonomic units (OTUs), and were dominated by Actinobacteria, Bacteriodetes, Cyanobacteria, and Proteobacteria (Figure 1). Temporal trends were detected for several bacteria phyla and classes, highlighted by an increasing proportion of Cyanobacteria and a decreasing proportion of Actinobacteria from May through August. Replicate samples were very similar in composition, except for surface water samples collected July 9, with replicate S1 having a notably higher percentage of Cyanobacteria than replicate S2. Shannon diversity ranged from 3.4 to 4.2 with a trend toward increased diversity over time, except for one replicate on July 9 sample (Figure 2). The Chao1 estimate of taxonomic richness changed little over time with the exceptions of high Chao1 in surface waters during the large precipitation event on May 1, and low Chao1 in surface waters on August 22 following a 10-day period of less than 0.01 inches of precipitation total (Figure 2).

**Figure 3:**
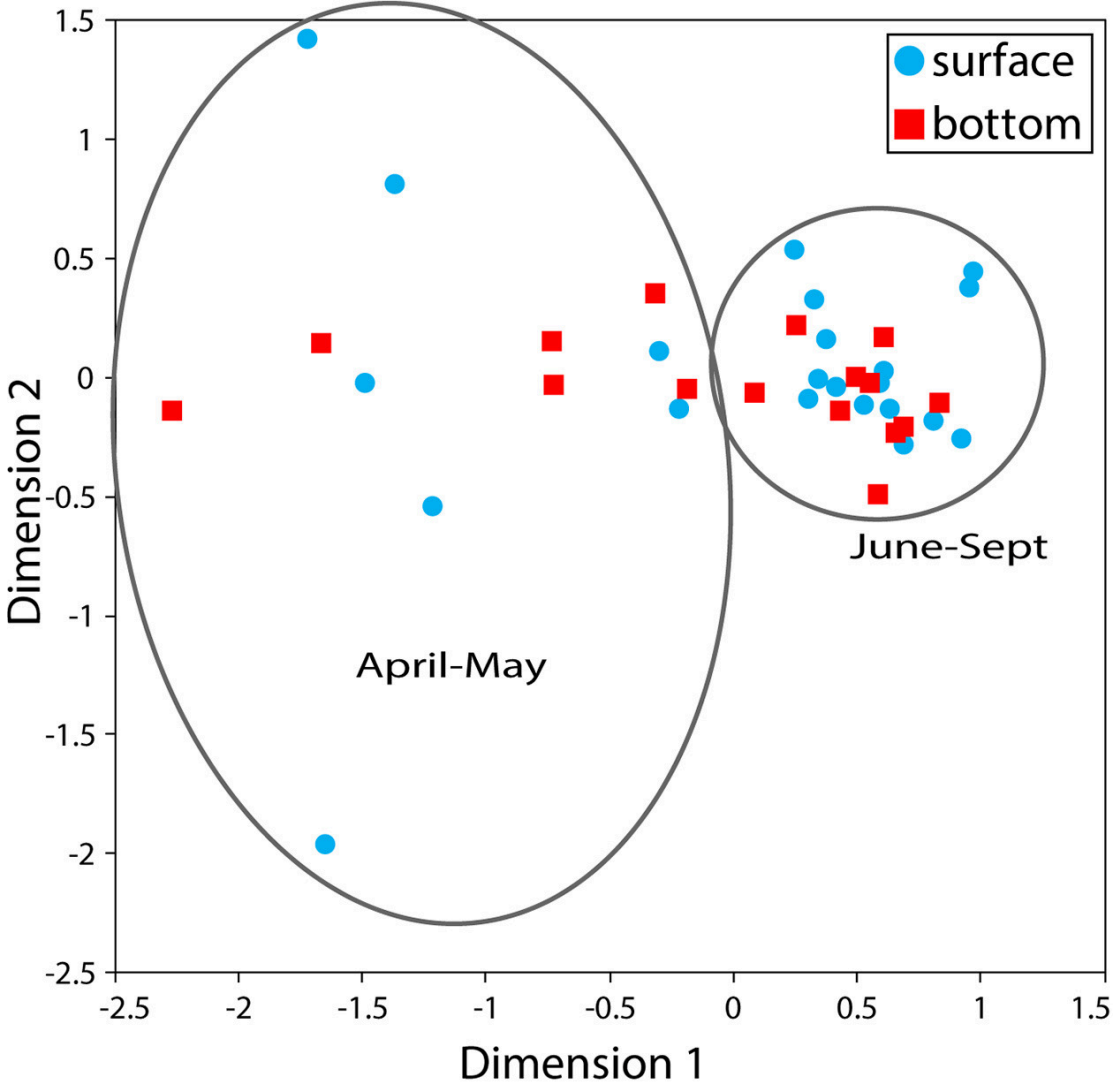
nMDS plot of beta diversity using Bray-Curtis similarity comparisons of log transformed 16S rRNA copy abundance. Surface nMDS plot based on 25 iterations with a final stress level of 0.01. Surface community composition (blue circles) showed higher variability in spring than in summer of fall.

Patterns in betadiversity showed that bacterial community composition was more variable in April–May, and relatively consistent in June–September (Figure 3). Bacterial community composition was significantly related to water temperature, turbidity, and salinity, which explained 29, 19, and 14% of variability in bacterial community composition, respectively (Table 1). Dissolved oxygen, wind direction, and rain in the previous 24 h were also significantly related to community composition, but they explained less of the variability (Table 1). Other variables (tide state, wind speed, cloud cover) were not significantly related bacterial community composition.

**Table 1:** Associations between environmental variables and patterns of community composition, as determined by distance-based linear modeling (DistLM).

**Variable**	**Pseudo-F**	***p*-value**	**Proportion**
Water Temp	14.80	**0.001**	0.291
Secchi Depth	8.19	**0.001**	0.185
Salinity	5.89	**0.003**	0.141
Dissolved Oxygen	3.78	**0.002**	0.095
Wind Direction	3.59	**0.006**	0.091
Rain in Last 24hrs	2.16	**0.038**	0.057
Tide State	1.62	0.096	0.043
Wind Speed	1.61	0.095	0.043
Cloud Cover	1.11	0.257	0.030

*Statistically significant p-values are in bold. Proportion is the proportion of variability explained by that parameter*.

### Internal control recovery and estimation of 16S rRNA gene abundances

**Figure 4:**
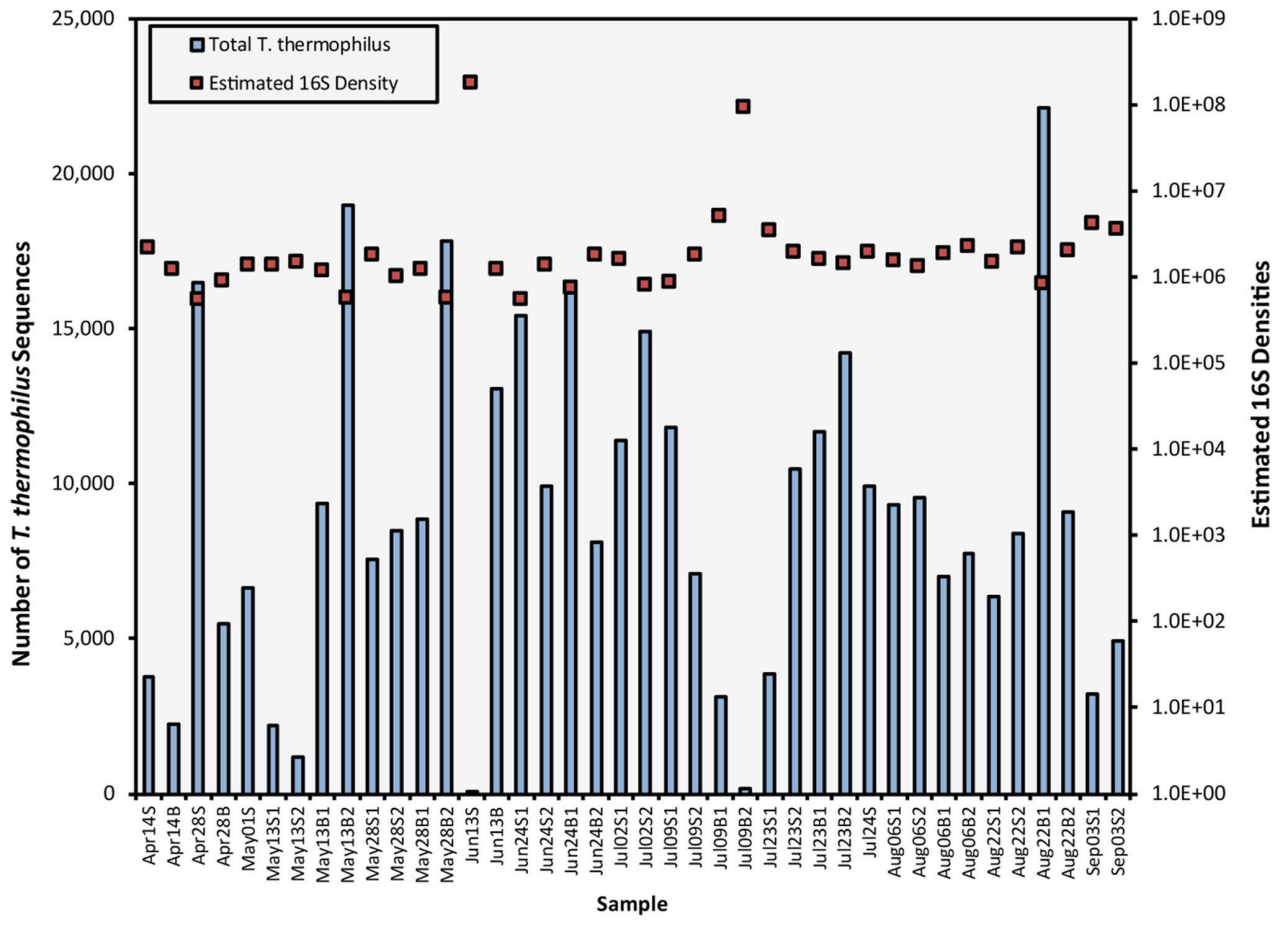
Comparison of the number of *T. thermophilus* (*Tt*) 16S rRNA reads (blue bars) and the estimated total 16S rRNA gene densities (#/mL) (red squares) for the 40 samples in this study. Sample names include month and day followed by S (surface) or B (bottom) and replicate number. Jun13S and Jul09B2 had unusually low numbers of *Tt* reads, indicating an unusually high density of bacteria or some error in the addition of *Tt* genes to those samples.

After removing unclassified sequences and those classified as Eukaryota, mitochondria, and chloroplasts, the 16S rRNA dataset contained 5,190,588 reads classified to 1,429 phylotypic OTUs. Converting the read counts to estimates of 16S rRNA gene abundances produced similar total 16S rRNA gene densities across samples (Figure 4). Despite notable variability in *T. thermophilus* sequence numbers between samples, all but two samples were estimated as having between 5.4 × 10^5^ and 5.1 × 10^6^ 16S genes/mL. These two samples (Jun13S and Jul09B2), contained very few *T. thermophilus* sequences, resulting in very high 16S gene abundances, and thus were eliminated from further, density-based analyses. The estimated abundance of 16S rRNA genes is similar to findings from other studies of Chesapeake Bay bacterial communities that have been measured as ranging from 2.0 to 24 × 10^6^ cells/mL (Shiah and Ducklow, 1994; Heidelberg et al., 2002).

### Genera with pathogenic members

**Figure 5:**
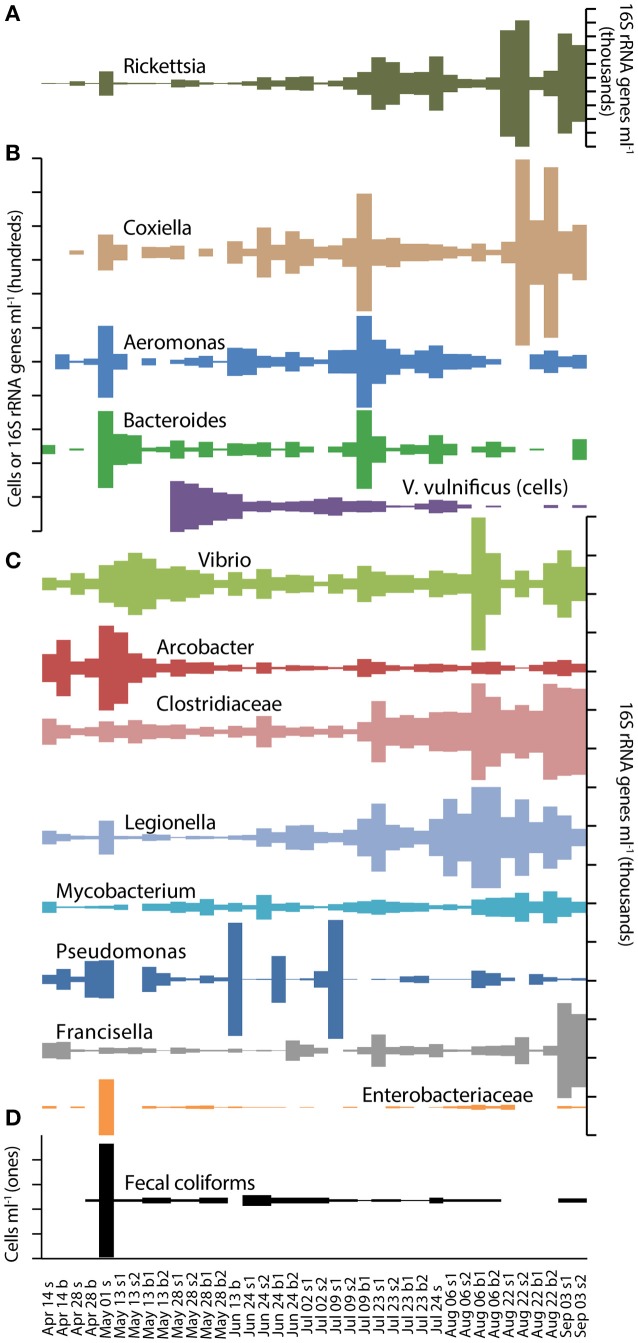
Abundances of *V. vulnificus* cells (qPCR), pathogen-containing phylogenetic groups (16S amplicons), and fecal coliforms (culturing) on scales of **(A)** thousands per ml, **(B)** hundreds per ml, **(C)** thousands per ml, and **(D)** individuals per ml on each sampling date.

A number of bacterial genera that include human pathogens were detected in the 16S rRNA gene sequences. All 38 samples contained members of the genus *Vibrio, Arcobacter* (Epsilonproteobacteria) spp., *Clostridium* spp. (Firmicutes), and *Legionella* spp. (Gammaproteobacteria) (Figure 5, Table 2), and most samples contained members of *Pseudomonas* spp. (Gammaproteobacteria), *Aeromonas* spp. (Gammaproteobacteria) and *Mycobacterium* spp. (Actinobacteria). Bacteria were also detected from 11 genera of the family Enterobacteriaceae, which includes most genera considered fecal coliform bacteria. *Enterococcus* spp. were detected in two samples (Table 2). The 16S rRNA gene densities for a number of individual Enterobacteriaceae groups as well as other potential pathogen containing groups, including *Aeromonas, Arcobacter, Bacteroides, Legionella*, and several genera of *Clostridium* were notably elevated or were exclusively detected on May 1 (Figure 5, Table 2). Bacterial groups not detected in these samples included *E. coli, Campylobacter, Salmonella, Listeria*, and *Helicobacter*.

**Table 2:** Occurrence of select bacterial groups that contain human pathogens.

**Family**	**Genus**	**Number of samples detected**	**1-May**		
			**Occurred**	**Elevated**	**Exclusive**
Aeromonadaceae	*Aeromonas*	31	✓		
Campylobacteraceae	*Arcobacter*	38	✓	✓	
Clostridiaceae	*Clostridium_sensu_stricto*	4			
	*Clostridium_sensu_stricto_1*	38	✓		
	*Clostridium_sensu_stricto_2*	6	✓		
	*Clostridium_sensu_stricto_3*	1	✓	✓	✓
	*Clostridium_sensu_stricto_5*	3	✓	✓	
	*Clostridium_sensu_stricto_7*	6			
	*Clostridium_sensu_stricto_8*	2			
	*Clostridium_sensu_stricto_9*	1			
	*Clostridium_sensu_stricto_10*	3	✓		
	*Clostridium_sensu_stricto_11*	3			
	*Clostridium_sensu_stricto_12*	9	✓		
	*Clostridium_sensu_stricto_13*	16	✓		
	*Clostridium_sensu_stricto_14*	2			
	*Clostridium_sensu_stricto_17*	17			
	*Clostridium_sensu_stricto_18*	7			
	*Clostridium_sensu_stricto_19*	1			
Coxiellaceae	*Coxiella*	32	✓		
Enterobacteriaceae	*Brenneria*	1	✓	✓	✓
	*Citrobacter*	3			
	*Enterobacter*	1			
	*Erwinia*	2	✓	✓	
	*Klebsiella*	1	✓	✓	✓
	*Morganella*	2			
	*Plesiomonas*	3			
	*Providencia*	1			
	*Rahnella*	2	✓	✓	
	*Serratia*	2	✓	✓	
	*Yersinia*	1	✓	✓	✓
Enterococcaceae	*Enterococcus*	2			
Francisellaceae	*Francisella*	37	✓		
Legionellaceae	*Legionella*	38	✓		
Leptospiraceae	*Leptospira*	11	✓		
Moraxellaceae	*Acinetobacter*	14	✓		
Mycobacteriaceae	*Mycobacterium*	37	✓		
Pseudomonadaceae	*Pseudomonas*	34	✓		
Rickettsiaceae	*Rickettsia*	38	✓		
Staphylococcaceae	*Staphylococcus*	9	✓		
Vibrionaceae	*Vibrio*	38	✓		

*The occurrence of pathogens on May 1, following the largest rain event, is highlighted by noting those pathogens that occurred on that date (Occurred), those that were at their highest density (Elevated), and those that were only detected on May 1 (Exclusive)*.

**Figure 6:**
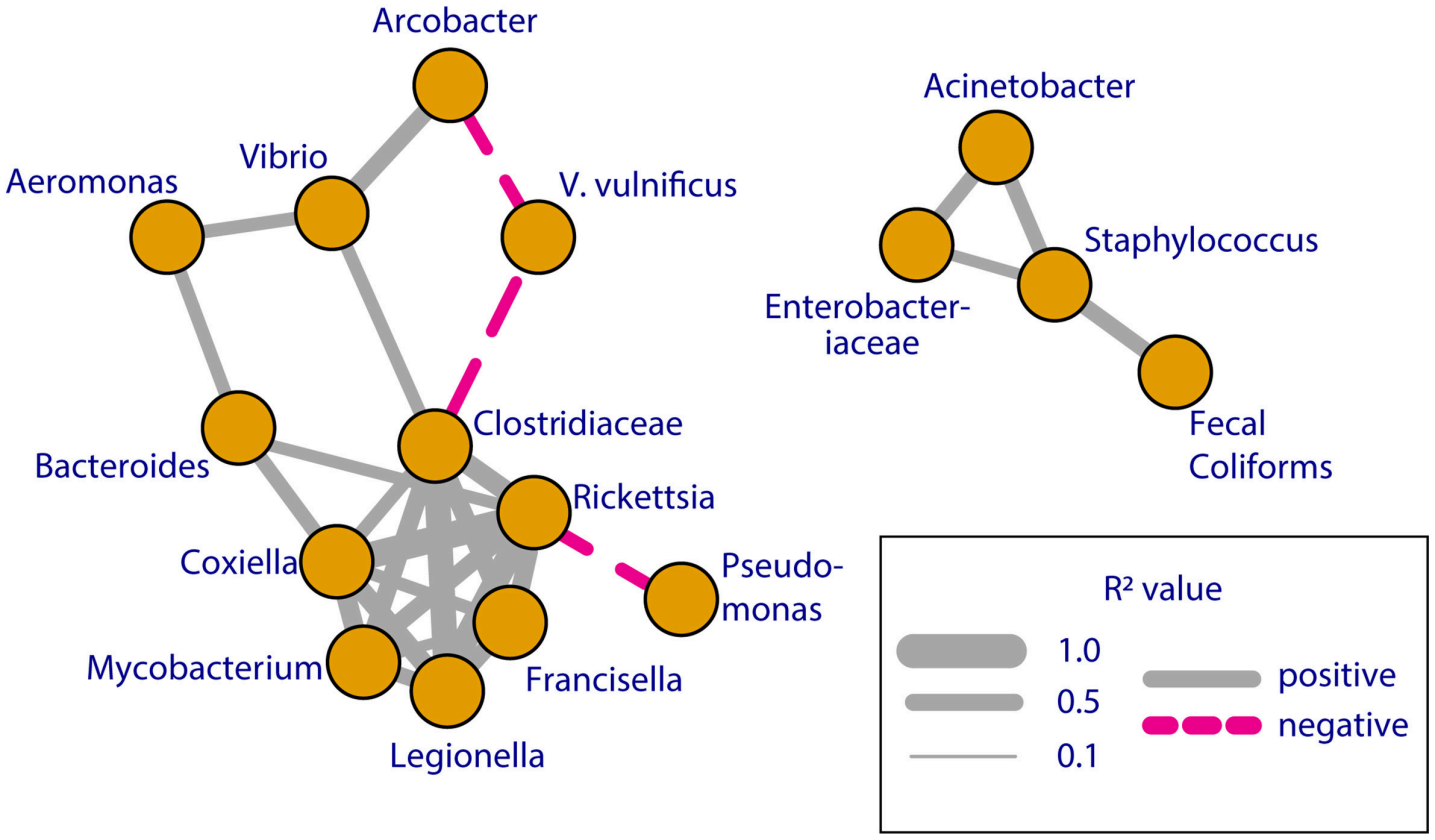
Correlation network of select pathogens (16S rRNA), fecal coliforms (culture), and *V. vulnificus* (qPCR) found in three or more samples. Connections represent significant (*p* < 0.05) Spearman rank correlations between pairs of taxa. Line thickness reflects the strength of this relationship (*R*^2^-value) and line color reflects whether the relationship was positive (solid, gray) or negative (dashed, red). All taxa included were correlated to at least one other taxa (Table S3). Line distance is not reflective of strength of correlation.

Significant correlations (Spearman rank correlation, *p* < 0.05) were detected between the abundances of pathogen containing groups, fecal coliforms and *Vibrio vulnificus* (Figure 6, Table S3). These groups fall into two clusters, which show the allochthonous community members correlating with fecal coliforms, and the more persistent members forming a separate cluster with *Vibrio* spp. The abundance of *V. vulnificus* was negatively correlated with two members of this second cluster, reflecting the persistent nature of *V. vulnificus* but its inverse abundance compared to these two other pathogens (Figure 5).

Several significant correlations (*p* < 0.05) occurred between the abundances of potential pathogen OTUs and environmental factors (Table S4). Clostridiaceae, *Legionella*, and Mycobacterium were positively correlated with the day of the year (DOY). Bacteroides and *Legionella* were positively correlated to water temperature, and several taxa were correlated with water temperature and Secchi depth, although some relationships were negative and others positive.

### Fecal coliform bacteria and *Vibrio* species

Fecal coliforms were detected on 11 of 14 sampling dates in both surface and bottom waters using standard culture methods (Figure 5C). All samples contained less than 0.5 cells per mL except on May 1 when fecal coliform abundance spiked to 4.5 cells/mL during the largest rain event. In contrast, samples collected on May 28, June 13 and July 24 contained relatively low fecal coliform densities, despite occurring soon after smaller rain events. Fecal coliforms were positively correlated with *Staphylococcus* 16S rRNA gene abundance, but not with any other potential pathogen OTUs (Table S3).

*V. vulnificus* was detected by qPCR assay in most samples after May 13 (Figure 5A), with greater than 100 cells per mL in the May 28 surface samples. *V. vulnificus* densities declined in mid-summer, and then increased in bottom water samples on August 6. *V. vulnificus* abundance did not correlate with *Vibrio* 16S rRNA gene abundance (Table S3). *Vibrio parahaemolyticus* was only detected in four samples with a maximum abundance of 4.7 cells/mL in one of the August 6 samples.

### Detection of potential and existing indicators

Several bacterial genera belonging to families that have been proposed as indicators of fecal pollution, several families of the order Bacteroidales and the family Lachnospiraceae, peaked in abundance on May 1 (Table 3), concurrent with a number of potential pathogen groups (Table 2).

**Table 3:** The occurrence of potential (Bacteroidales and Clostridiales) and existing (Enterococcus) fecal indicator groups, the number of samples (out of 38) in which they occurred, those that were detected on May 1 (Occurred), those that were elevated on May 1 at least 2 times the standard deviation above their mean (Elevated), and those that were only detected on May 1 (Exclusive).

**Order**	**Family**	**Genus**	**Number of Samples**	**Post Rain Event**		
				**Occurred**	**Elevated**	**Exclusive**
Bacteroidales	Bacteroidaceae	*Bacteroides*	33	✓	✓	
	Marinilabiaceae	*Mangroviflexus*	2	✓	✓	
		*Marinifilum*	14	✓		
	Porphyromonadaceae	*Dysgonomonas*	1	✓	✓	✓
		*Macellibacteroides*	2	✓	✓	
		*Paludibacter*	16	✓	✓	
		*Parabacteroides*	2	✓	✓	
	Prevotellaceae	*Prevotella*	6	✓	✓	
	S24-7	unclassified	4	✓	✓	
	unclassified	unclassified	19	✓	✓	
Clostridiales	Lachnospiraceae	*Anaerosporobacter*	3	✓	✓	
Lactobacillales	Enterococcaceae	*Enterococcus*	2			

## Discussion

Deep amplicon sequencing of 16S rRNA genes at the shellfish monitoring station examined in this study identified many pathogen-containing bacterial families and genera that were present throughout the spring and summer. Other pathogen containing groups identified by 16S rRNA sequencing were greatly elevated in abundance and diversity on May 1 following the only rainstorm that exceeded 1 inch in the previous 24 h. Fecal coliforms were also in high abundance after this rainstorm, and, over time, their abundance correlated with the abundance of *Staphylococcus* sp. 16S genes. In contrast, *V. vulnificus* (quantified via qPCR) were absent after this rainstorm, and did not appear until May 28 when water temperature increased to 24.2°C and salinity decreased to 9.3 ppt. *V. vulnificus* were detected in most samples after May 28, but did not correlate with fecal coliform abundance, *Vibrio* sp. 16S rRNA genes, or with any other taxa except for weak negative correlations with *Arcobacter* sp. and *Pseudomonas* sp. 16S rRNA genes. Thus, while 16S rRNA gene amplicon sequencing detected potential pathogens in Chesapeake Bay waters, elevated abundances of some of these organisms only corresponded to elevated abundances of the fecal coliform indicator method following a major rainstorm. These results demonstrate that many potential pathogens co-occur with high densities of fecal coliform bacteria following large storms, but are poorly correlated at other times, and do not include autochthonous potential pathogens like *V. vulnificus*.

Changes in the broader bacterial community over time correlated with seasonal changes in environmental factors including water temperature, Secchi depth, dissolved oxygen, and salinity. Similar seasonal patterns have been seen in other estuaries (Crump et al., 2003; Fortunato and Crump, 2011; Pereira et al., 2015), and are likely driven by species sorting of microbial communities in order to adapt to multiple environmental factors that change as seasons shift from spring to summer to fall. This is consistent with Kan et al. (2006) who found that seasonal variability in bacterial communities at any particular location in Chesapeake Bay far exceeded spatial variability across the entire salinity gradient of Chesapeake Bay. Kan et al. (2006) found that this variability was most strongly correlated with seasonal changes in temperature and chlorophyll *a* concentration, matching well with the top environmental correlates from the shellfish monitoring station (temperature and Secchi depth). This suggests that the microbial community at the shellfish monitoring station is representative of microbial communities throughout the Chesapeake Bay, and that variability driven by inputs of allochthonous organisms from land is infrequent and temporary.

The greatest change in community composition at the monitoring station occurred after a 24-h rainfall of 1.3 inches, and featured a decrease in typical planktonic taxa (Actinobacteria, Gammaproteobacteria), and an increase in Bacteroidetes, Cyanobacteria, and several taxa typical of soils (Acidobacteria, Firmicutes). Alpha diversity of the bacterial community was also highest on that day, further supporting the idea that this rainfall introduced allochthonous (i.e., non-endemic) bacteria to the community. This suggests that runoff from land can influence the chance of encountering pathogen-containing phylogenetic groups in nearshore water, but only for short periods of time. It also suggests a nonlinear relationship between precipitation and the bacterial community, with some threshold below 1.3 inches of rain capable of causing a distinct shift in the community. Salinity at the site was lowered by 1.4 ppt following this storm, suggesting a 13% dilution with rainwater. This level of dilution was not seen following any of the smaller storms.

Trends in the abundance of bacterial taxa of concern to shellfish harvest managers followed two general patterns that appear to distinguish between autochthonous (endemic) bacteria and allochthonous (land-based or benthic) bacteria. *Mycobacterium, Legionella*, and Clostridiaceae are autochthonous taxa commonly found in fresh and estuarine waters, with densities driven by water conditions like temperature and salinity (Grimes, 1991). In samples where they were detected, 16S rRNA gene abundances for many of these groups were positively correlated with each other, and were positively correlated with temperature and day of the year. Similarly, the abundances of potential pathogen groups typically associated with runoff and land-based sources (Ferguson et al., 1996; Ackerman and Weisberg, 2003), such as Enterobacteriaceae, *Aeromonas, Arcobacter* and *Bacteroides* did not correlate with the autochthonous groups but some correlated with each other. However, few of these allochthonous taxa correlated positively with temperature because they spiked in abundance in cooler water following the large rain event on May 1, and several were also abundant in bottom waters on July 9. *Vibrio* sp. abundance was correlated with members of both of these groups of taxa, including Clostridiaceae and *Arcobacter*, likely reflecting the highly variable characteristics and life cycles of diverse *Vibrio* species (Thompson et al., 2004). Several pathogen groups, such as *Salmonella, Campylobacter*, and *Listeria* were never detected, despite previous evidence of the presence of some of these groups in Chesapeake Bay waters (Sayler et al., 1975) and shellfish meat (Rawles et al., 1995). The detection of *Coxiella, Francisella*, and *Rickettsia* bacteria in most samples despite their lack of association with waterborne diseases advocates for the monitoring of these pathogen groups in natural water bodies.

The occurrence of both autochthonous and allochthonous pathogen-containing genera underscores the need for a management strategy capable of assessing potential pathogens from multiple sources in shellfish and recreational waters. For example, the appearance or increase in abundance of several members of the groups Enterobacteriaceae, *Acrobacter, Aeromonas*, and *Bacteroides*, and their co-occurrence with a spike in culture-based fecal coliform densities supports the use of these indicators as criteria for closing shellfish beds to harvest (FDA, 2013). Also, the co-occurrence of several genera of Bacteriodales and Lachnospiraceae with pathogen-containing groups encourages further study into using these families as alternative indicators of human waste (Bernhard and Field, 2000; Newton et al., 2011). However, a different strategy is required for pathogen groups that are not related to precipitation. The ubiquity of some of these groups, such as *V. vulnificus*, is known (Wright et al., 1996; Jacobs et al., 2014), particularly in summer months. The detection of *Vibrio, Pseudomonas, Legionella, Mycobacterium*, and Clostridiaceae 16S rRNA genes in most of the samples is also consistent with their ubiquitous presence in aquatic environments. The strategy for assessing the presence and/or abundance of these endemic pathogen-containing groups may rely upon qPCR detection in shellfish tissues (Brasher et al., 1998) or the use of spatially-explicit predictive models (Jacobs et al., 2014). The challenge to the management community is to discover and implement cost-effective monitoring tools that will more accurately assess the relative risk of encountering both allochthonous and autochthonous pathogens.

Our 16S rRNA gene classification of the bacterial community at a shellfish monitoring station identified a broad range of potential pathogen groups that may serve as useful indicators of poor water quality. However, all of these taxonomic groups include both virulent and non-virulent strains, suggesting that it is necessary to develop procedures that target species-specific genes and virulence genes from virulent members of these groups in order to make management decisions about water quality in shellfish growing areas. Although virulence factors are complex and subject to evolution and transfer between microbes (Hacker et al., 1997), future study of their occurrence, coupled with 16S rRNA gene diversity studies, in shellfish waters may improve our assessment of risks to human health.

Using an internal control to normalize sequences across samples and to estimate 16S rRNA gene densities is a powerful new approach for microbial ecology (Satinsky et al., 2013; Smets et al., 2016), because it provides per liter abundances of individual microbial taxa for comparison with qPCR and culture-based estimates. Estimates of total bacterial 16S rRNA genes in our samples were within the range of values for abundance of total bacterial cells in estuarine waters (Ducklow, 1982). However, the number of 16S rRNA genes per bacterial cell varies between 1 and 15 (Kembel et al., 2012), with greater numbers typical of copiotrophic bacteria that maintain large amounts of genetic material in order to quickly take advantage of nutrient introductions (Klappenbach et al., 2000). Although methods for estimating bacterial cell abundance based on 16S rRNA gene copies have been developed (Kembel et al., 2012), there are still large groups of uncultured bacteria for which the number of 16S rRNA genes per cell are unknown. This approach may be strengthened in future studies by making additional estimates of cell densities from staining and direct counting of total bacterial cells, from fluorescence in-situ hybridization using probes for specific groups, and from qPCR methods.

Although the use of an internal control to estimate the abundance of 16S rRNA genes in environmental samples shows some promise, differences in abundance estimates between 16S sequencing and the culture and qPCR-based methods suggest that there are some unquantified biases in these techniques. Enterobacteriaceae (16S sequencing) and fecal coliform (culture) bacteria co-occurred, due primarily to the peak in abundance on May 1, but cultured fecal coliform abundances were two to three orders of magnitude lower than the 16S rRNA gene densities (Figure 5). This discrepancy is likely a result of the fact that the family Enterobacteriaceae includes members that are not considered fecal coliforms and that do not grow optimally at 44°C. Additionally, Enterobacteriaceae densities based on 16S rRNA gene data may include bacteria that were non-culturable and therefore not represented in the fecal coliform counts, reflecting the well-known difficulties in quantitatively culturing bacteria (Staley and Konopka, 1985). Some of this discrepancy could also partially be explained if naturally-occurring Enterobacteriaceae have multiple 16S genes per genome (Stoddard et al., 2015).

Total *Vibrio* 16S rRNA gene abundance was up to approximately 500 times greater than *V. vulnificus* abundance estimated by qPCR (Aug 6 bottom sample), and averaged approximately 30 times greater. However, nine samples contained *V. vulnificus* estimates greater than the total *Vibrio* (16S) estimate suggesting that, for these samples, either the qPCR technique overestimated *V. vulnificus* abundance or amplicon sequencing underestimated *Vibrio* spp. 16S gene abundance. The qPCR method quantifies *V. vulnificus* by comparing sample data to a standard curve prepared with known densities of cultured *V. vulnificus* cells. This method could overestimate *V. vulnificus* if the copy number of genes targeted by qPCR is elevated in natural populations of *V. vulnificus* compared to the cultured representatives. It is also possible that the PCR used to amplify 16S rRNA genes prior to sequencing under-amplified *Vibrio* sp. 16S rRNA genes, although the 16S rRNA PCR primers used for this study exactly match *T. thermophilus* and all Enterobacteriaceae and *Vibrio* taxa in the Silva database (v1.19). However, PCR bias may have affected estimates of 16S rRNA genes due to differences in the secondary structure and the G:C content of the various amplicons (Suzuki and Giovannoni, 1996). Pinto and Raskin (2012) showed that, although increased sequencing depth corresponded to improved taxa detection frequency, amplicon structure can affect the number of reads. Suzuki et al. (1998) also showed that over-amplification of genes in a mixed bacterial community can cause over-representation of rare taxa and underrepresentation of more common taxa, but in this study, *Vibrio* would be considered rare and therefore unlikely to be under-amplified. An important next step in using *T. thermophilus* or similar internal control approaches for estimating cell abundances is the development of a quantitative PCR assay to assess the recovery of the internal control after sample extraction.

Despite these discrepancies, 16S rRNA gene amplicon sequencing allowed a comprehensive examination of bacterial communities in Chesapeake Bay waters, providing information useful in assessing potential human health risks associated with exposures to this water body. For example, *Acinetobacter* bacteria, detected in 15 of 38 samples, is a genus of bacteria found in soil and water at low densities (Baumann, 1968). Some members of this genus are known to express multi-drug resistance and virulence factors (Towner, 2009). Moreover, Zhang et al. (2009) showed that certain wastewater treatment processes may select for multi-drug-resistant *Acinetobacter* strains in receiving waters. *Dysgonomonas*, which were present on May 1, are much less common in aquatic environments, but are also known for multi-drug resistance and pathogenicity (Vaughan and Forbes, 2014). As sequencing methods produce larger numbers of reads per sample, and as read classification improves with expanding phylogenetic databases, assessments of bacterial communities and associated pathogens in shellfish growing and recreational-use areas will improve our understanding of the ecology of these bacterial groups and how to best manage human health risks.

Several conclusions may be drawn from this study. Significant changes in bacterial community composition, including fecal bacteria and fecal indicators may occur from single rain events with a threshold of precipitation occurring at this sampling location. Water temperature may also influence bacterial community composition over time. PCR quantification of *V. vulnificus* and *V. parahaemolyticus* showed that only a subset of samples contained these pathogens at densities above the limit of detection and that their abundances did not correlate with the ubiquitous *Vibrio* 16S rRNA gene densities. If coupled with virulence information, 16S rRNA gene sequencing studies may provide useful information regarding the types, amounts, and temporal dynamics of pathogenic bacteria that occur in managed waters and be used to inform decisions about seafood safety.

## Author contributions

AL, BC, and RH conceived and designed the study; AL performed sample collection and processing; AL and BC performed data analysis; AL, BC, and RH drafted and critically revised the paper. All authors read and approved the final version of the manuscript.

### Conflict of interest statement

The authors declare that the research was conducted in the absence of any commercial or financial relationships that could be construed as a potential conflict of interest.
