# Supplementary material for: Assessment of Fecal Indicator Bacteria and Potential Pathogen Co-Occurrence at a Shellfish Growing Area
Source: Front Microbiol. 2018 Mar 14;9:384. doi: 10.3389/fmicb.2018.00384 (PMC5861211; doi:10.3389/fmicb.2018.00384)
Supplement: Supplementary file 1 [file Table1.docx]

***Supplementary Material***

**Assessment of fecal indicator bacteria and potential pathogen co-occurrence at a shellfish growing area**

**Andrew K. Leight^1,2*^, Byron C. Crump^3^, Raleigh R. Hood^2^**

**Correspondence:** Andrew Leight [ak.leight@noaa.gov](mailto:ak.leight@noaa.gov)

Figure S1. Location of sampling station and rain gauge.
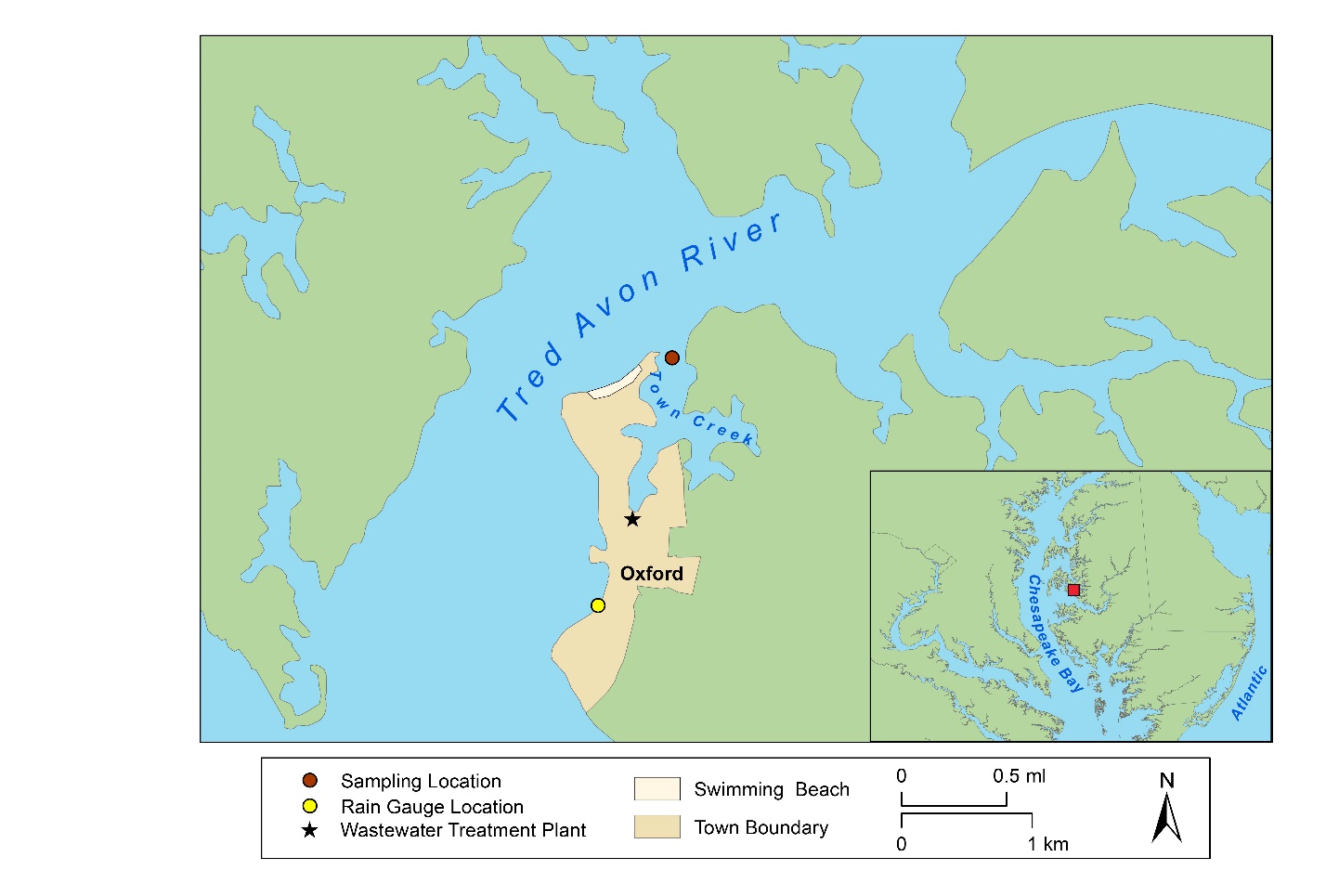


Table S1. Real-time PCR primers and probes used to detect and quantify select *Vibrio* species.

| Bacteria | Reference(s) | Gene Target | Primer or Probe | Sequence (5’-3’) |
| --- | --- | --- | --- | --- |
| *V. vulnificus* | Panicker and Bej, 2005; Jacobs et al, 2014 | hemolysin a | *Vvh* forward | TTCCAACTTCA AACCGAACTATGA |
|  |  |  | *Vvh* reverse | TTCCAGTCGATGCGAATACGTTG |
|  |  |  | *Vvh*874 probe | 56-FAM/AACTATCGTGCACGCTTTGGTACCGT/3IABkFQ/ |
| *V. parahaemolyticus* | Nordstrom et al, 2007 | thermo-labile hemolysin | *tlh* forward | ACTCAACACAAGAAGAGATCGACAA |
|  |  |  | *tlh* reverse | GATGAGCGGTTGATGTCCAA |
|  |  |  | *tlh* probe | 56-FAM/CGCTCGCGTTCACGAAACCGT/BHQ2 |

Table S2. Physical and chemical characteristics of surface and bottom waters at the sampling location. Dissolved oxygen data represents as a percent of saturation (temperature adjusted). Surface samples were collected approximately 20 cm below the surface and bottom samples were collected at a depth of approximately 1.7 m.

| **Date** | **Time** | **Depth** | **Water Temp (°C)** | **Salinity (ppt)** | **pH** | **Dissolved Oxygen (%)** | **Secchi Depth (m)** |
| --- | --- | --- | --- | --- | --- | --- | --- |
| 4/14/2014 | 9:00 | Surface | 15.29 | 10.93 | 7.19 | 100.4 | 0.7 |
| 4/14/2014 | 9:00 | Bottom | 15.36 | 10.97 | 7.15 | 98.1 | 0.7 |
| 4/28/2014 | 9:30 | Surface | 15.35 | 11.74 | 7.39 | 112.3 | 1 |
| 4/28/2014 | 9:30 | Bottom | 15.35 | 11.62 | 7.38 | 112.3 | 1 |
| 5/1/2014 | 8:30 | Surface | 15.12 | 10.24 | 7.88 | 105.1 | 0.9 |
| 5/1/2014 | 8:30 | Bottom | 14.75 | 10.62 | 7.87 | 102.2 | 0.9 |
| 5/13/2014 | 9:45 | Surface | 22.73 | 9.98 | 8.18 | 107.4 | 1.1 |
| 5/13/2014 | 9:45 | Bottom | 21.12 | 9.98 | 8.15 | 79.9 | 1.1 |
| 5/28/2014 | 9:49 | Surface | 24.22 | 9.31 | 8.13 | 79.30 | 0.8 |
| 5/28/2014 | 9:49 | Bottom | 23.91 | 9.30 | 8.09 | 66.10 | 0.8 |
| 6/13/2014 | 10:45 | Surface | 25.77 | 9.11 | 8.25 | 88.3 | 0.7 |
| 6/13/2014 | 10:45 | Bottom | 25.08 | 9.13 | 8.27 | 84.9 | 0.7 |
| 6/24/2014 | 9:15 | Surface | 25.94 | 9.13 | 7.97 | 85.2 | 0.8 |
| 6/24/2014 | 9:15 | Bottom | 25.77 | 9.11 | 8.07 | 81.2 | 0.8 |
| 7/2/2014 | 10:00 | Surface | 28.01 | 9.26 | 8.14 | 93.2 | 0.7 |
| 7/2/2014 | 10:00 | Bottom | 27.82 | 9.25 | 8.15 | 87.7 | 0.7 |
| 7/9/2014 | 10:05 | Surface | 27.43 | 9.3 | 8.26 | 100.8 | 0.6 |
| 7/9/2014 | 10:05 | Bottom | 27.1 | 9.3 | 8.29 | 93.3 | 0.6 |
| 7/23/2014 | 8:55 | Surface | 27.56 | 9.31 | 8.1 | 87.6 | 0.55 |
| 7/23/2014 | 8:55 | Bottom | 27.18 | 9.28 | 8.13 | 84.3 | 0.55 |
| 7/24/2014 | 9:15 | Surface | 27.85 | 9.21 | 8.08 | 82 | 0.6 |
| 7/24/2014 | 9:15 | Bottom | 27.84 | 9.22 | 8.11 | 79.6 | 0.6 |
| 8/6/2014 | 8:50 | Surface | 26.42 | 9.41 | 8.02 | 94.10 | 0.7 |
| 8/6/2014 | 8:50 | Bottom | 26.25 | 9.41 | 8.04 | 84.70 | 0.7 |
| 8/22/2014 | 9:30 | Surface | 26.85 | 10.02 | 8.08 | 82.30 | 0.8 |
| 8/22/2014 | 9:30 | Bottom | 26.65 | 10.02 | 8.09 | 65.90 | 0.8 |
| 9/3/2014 | 8:49 | Surface | 27.30 | 10.87 | 8.23 | 93.20 | 0.7 |
| 9/3/2014 | 8:49 | Bottom | 27.22 | 10.87 | 8.29 | 92.80 | 0.7 |


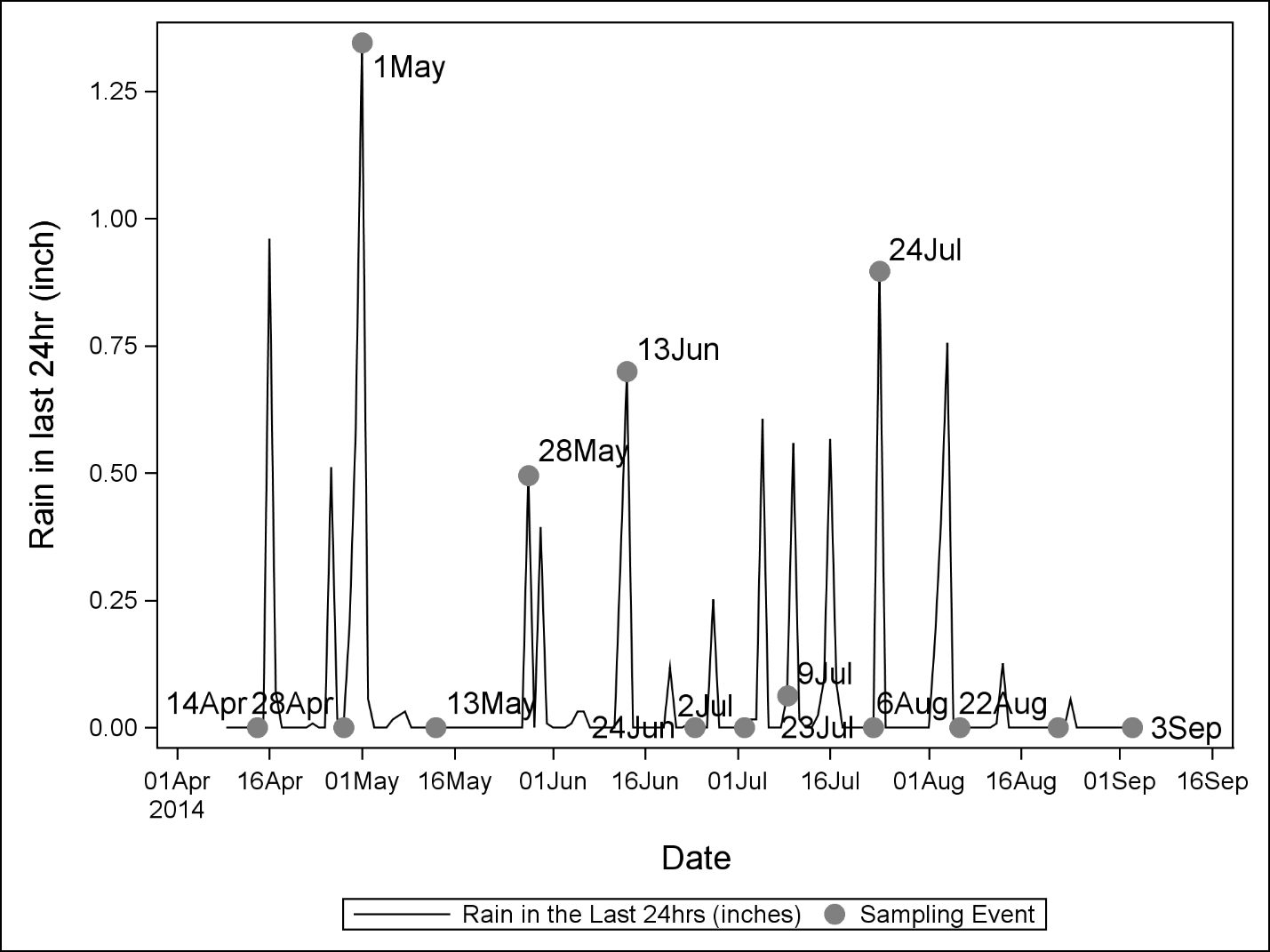


Figure S2. Total rain (inches) in the 24 hours preceding sampling for each sample collection date (gray dots). May 1 was the only sampling date with greater than 1 inch of rain, with three other sampling dates having 0.5 to 0.85 inches of rain preceding sampling.

Table S3. Spearman rank correlations among fecal coliform abundance (culture), *V. vulnificus* abundance (qPCR), and the abundance of pathogen-containing taxonomic groups (16S amplicon sequencing).

|  |  | Fecal Coliforms | *V. vulnificus* | *Acinetobacter* | *Aeromonas* | *Arcobacter* | *Bacteroides* | Clostridiaceae | *Coxiella* | Entero- bacteriaceae | *Francisella* | *Legionella* | *Mycobacterium* | *Pseudomonas* | *Rickettsia* | *Staphylococcus* |
| --- | --- | --- | --- | --- | --- | --- | --- | --- | --- | --- | --- | --- | --- | --- | --- | --- |
| *V. vulnificus* | R | 0.23 |  |  |  |  |  |  |  |  |  |  |  |  |  |  |
|  | s | 0.190 |  |  |  |  |  |  |  |  |  |  |  |  |  |  |
| *Acinetobacter* | R | -0.04 | 0.23 |  |  |  |  |  |  |  |  |  |  |  |  |  |
|  | s | 0.845 | 0.167 |  |  |  |  |  |  |  |  |  |  |  |  |  |
| *Aeromonas* | R | 0.09 | 0.24 | 0.15 |  |  |  |  |  |  |  |  |  |  |  |  |
|  | s | 0.629 | 0.154 | 0.363 |  |  |  |  |  |  |  |  |  |  |  |  |
| *Arcobacter* | R | -0.26 | **-0.36** | 0.08 | 0.30 |  |  |  |  |  |  |  |  |  |  |  |
|  | s | 0.144 | **0.027** | 0.613 | 0.065 |  |  |  |  |  |  |  |  |  |  |  |
| *Bacteroides* | R | 0.04 | 0.31 | -0.04 | **0.38** | -0.04 |  |  |  |  |  |  |  |  |  |  |
|  | s | 0.831 | 0.058 | 0.802 | **0.018** | 0.832 |  |  |  |  |  |  |  |  |  |  |
| Clostridiaceae | R | -0.16 | **-0.35** | -0.26 | -0.06 | 0.12 | 0.05 |  |  |  |  |  |  |  |  |  |
|  | s | 0.377 | **0.030** | 0.121 | 0.725 | 0.476 | 0.774 |  |  |  |  |  |  |  |  |  |
| *Coxiella* | R | 0.23 | 0.02 | -0.07 | 0.15 | -0.19 | **0.44** | **0.41** |  |  |  |  |  |  |  |  |
|  | s | 0.194 | 0.891 | 0.696 | 0.373 | 0.264 | **0.006** | **0.011** |  |  |  |  |  |  |  |  |
| Entero- | R | 0.30 | 0.29 | 0.45 | 0.15 | -0.05 | -0.03 | -0.09 | -0.14 |  |  |  |  |  |  |  |
| bacteriaceae | s | 0.093 | 0.073 | 0.004 | 0.367 | 0.785 | 0.844 | 0.591 | 0.386 |  |  |  |  |  |  |  |
| *Francisella* | R | -0.27 | -0.22 | -0.19 | 0.14 | 0.24 | 0.04 | **0.54** | **0.38** | -0.27 |  |  |  |  |  |  |
|  | s | 0.123 | 0.179 | 0.255 | 0.417 | 0.147 | 0.830 | **0.001** | **0.019** | 0.100 |  |  |  |  |  |  |
| *Legionella* | R | -0.04 | -0.18 | -0.22 | -0.02 | -0.14 | 0.28 | **0.75** | **0.57** | -0.16 | **0.51** |  |  |  |  |  |
|  | s | 0.826 | 0.285 | 0.178 | 0.916 | 0.402 | 0.092 | **<.0001** | **<.0001** | 0.344 | **0.001** |  |  |  |  |  |
| *Mycobacterium* | R | -0.02 | 0.06 | -0.02 | 0.05 | -0.05 | 0.00 | **0.53** | **0.57** | 0.05 | **0.32** | **0.46** |  |  |  |  |
|  | s | 0.918 | 0.707 | 0.882 | 0.772 | 0.760 | 0.984 | **0.001** | **<.0001** | 0.764 | **0.047** | **0.003** |  |  |  |  |
| *Pseudomonas* | R | 0.04 | -0.09 | 0.26 | -0.28 | 0.10 | -0.15 | -0.05 | -0.25 | 0.21 | -0.27 | -0.20 | -0.13 |  |  |  |
|  | s | 0.829 | 0.598 | 0.108 | 0.090 | 0.557 | 0.358 | 0.765 | 0.138 | 0.196 | 0.100 | 0.237 | 0.429 |  |  |  |
| *Rickettsia* | R | 0.07 | 0.06 | -0.10 | 0.15 | -0.24 | 0.36 | **0.60** | **0.77** | -0.03 | **0.54** | **0.72** | **0.52** | **-0.40** |  |  |
|  | s | 0.679 | 0.704 | 0.558 | 0.385 | 0.139 | 0.027 | **<.0001** | **<.0001** | 0.860 | **0.000** | **<.0001** | **0.001** | **0.014** |  |  |
| *Staphylococcus* | R | **0.48** | 0.16 | **0.44** | 0.09 | -0.27 | 0.06 | -0.23 | 0.12 | **0.34** | -0.17 | -0.02 | -0.01 | 0.07 | 0.13 |  |
|  | s | **0.005** | 0.335 | **0.006** | 0.583 | 0.101 | 0.709 | 0.160 | 0.473 | **0.035** | 0.310 | 0.898 | 0.967 | 0.667 | 0.432 |  |
| *Vibrio* | R | 0.06 | -0.11 | 0.00 | **0.39** | **0.52** | 0.01 | **0.38** | 0.21 | 0.01 | 0.25 | 0.21 | **0.28** | -0.16 | 0.18 | -0.14 |
|  | s | 0.758 | 0.513 | 0.990 | **0.016** | **0.001** | 0.935 | **0.019** | 0.210 | 0.936 | 0.133 | 0.209 | **0.083** | 0.333 | 0.280 | 0.399 |

Table S4. Spearman rank correlations among environmental parameters (day of the year (DOY)), water temperature, Secchi depth, and the total amount of rain in the last 24 hours) to fecal coliform abundance (culture), *V. vulnificus* abundance (qPCR), and the abundance of pathogen-containing taxonomic groups (16S amplicon sequencing).

|  |  | DOY | Water Temp | Salinity | Secchi Depth | Rain Last 24hr |
| --- | --- | --- | --- | --- | --- | --- |
| Water Temp | R | **0.77** |  |  |  |  |
|  | s | **<.0001** |  |  |  |  |
| Salinity | R | -0.07 | **-0.39** |  |  |  |
|  | s | 0.679 | **0.016** |  |  |  |
| Secchi Depth | R | **-0.49** | **-0.73** | **0.34** |  |  |
|  | s | **0.002** | **<.0001** | **0.039** |  |  |
| Rain Last 24hr | R | -0.22 | -0.08 | -0.27 | -0.08 |  |
|  | s | 0.194 | 0.648 | 0.095 | 0.644 |  |
| Fecal Coliforms | R | 0.01 | 0.06 | **-0.42** | **0.38** | 0.18 |
|  | s | 0.978 | 0.745 | **0.016** | **0.031** | 0.317 |
| *V. vulnificus* | R | 0.10 | **0.37** | **-0.73** | **-0.39** | **0.58** |
|  | s | 0.534 | **0.021** | **<.0001** | **0.016** | **<.0001** |
| *Acinetobacter* | R | **-0.33** | -0.28 | -0.08 | 0.10 | **0.44** |
|  | s | **0.042** | 0.087 | 0.626 | 0.562 | **0.005** |
| *Aeromonas* | R | -0.11 | 0.00 | -0.29 | -0.10 | **0.38** |
|  | s | 0.519 | 0.984 | 0.078 | 0.539 | **0.019** |
| *Arcobacter* | R | **-0.46** | **-0.56** | **0.54** | **0.32** | 0.07 |
|  | s | **0.004** | **<.0001** | **<.0001** | **0.049** | 0.686 |
| *Bacteroides* | R | 0.21 | **0.35** | **-0.36** | **-0.50** | 0.30 |
|  | s | 0.205 | **0.034** | **0.028** | **0.002** | 0.063 |
| Clostridiaceae | R | **0.67** | 0.27 | **0.37** | -0.26 | **-0.37** |
|  | s | **<.0001** | 0.097 | **0.021** | 0.117 | **0.023** |
| *Coxiella* | R | **0.61** | **0.54** | -0.13 | -0.26 | -0.03 |
|  | s | **<.0001** | **<.0001** | 0.454 | 0.113 | 0.851 |
| Entero- | R | -0.10 | -0.29 | -0.13 | 0.22 | 0.46 |
| bacteriaceae | s | 0.544 | 0.078 | 0.423 | 0.183 | 0.004 |
| *Francisella* | R | **0.36** | 0.32 | 0.25 | **-0.35** | -0.23 |
|  | s | **0.028** | 0.051 | 0.131 | **0.031** | 0.159 |
| *Legionella* | R | **0.75** | **0.52** | 0.09 | **-0.45** | -0.31 |
|  | s | **<.0001** | **0.001** | 0.590 | **0.005** | 0.058 |
| *Mycobacterium* | R | **0.56** | 0.29 | 0.01 | -0.21 | -0.07 |
|  | s | **<.0001** | 0.082 | 0.948 | 0.210 | 0.663 |
| *Pseudomonas* | R | -0.28 | -0.29 | 0.11 | 0.07 | 0.12 |
|  | s | 0.094 | 0.075 | 0.510 | 0.668 | 0.461 |
| *Rickettsia* | R | **0.79** | **0.68** | -0.04 | **-0.44** | -0.03 |
|  | s | **<.0001** | **<.0001** | 0.815 | **0.006** | 0.852 |
| *Staphylococcus* | R | -0.08 | -0.01 | **-0.32** | 0.17 | 0.11 |
|  | s | 0.647 | 0.975 | **0.049** | 0.298 | 0.494 |
| *Vibrio* | R | 0.11 | -0.13 | 0.21 | 0.21 | 0.08 |
|  | s | 0.529 | 0.421 | 0.209 | 0.198 | 0.644 |
